# The role of untranslated region variants in Mendelian disease: a review

**DOI:** 10.1038/s41431-025-01905-x

**Published:** 2025-07-03

**Authors:** Nechama Wieder, Elston N. D’Souza, Ruebena Dawes, Alexander Chan, Alexandra Martin-Geary, Nicola Whiffin

**Affiliations:** 1 Big Data Institute, University of Oxford, Oxford, UK; 2 Centre for Human Genetics, University of Oxford, Oxford, UK; 3 Department of Biomedical Engineering, University of Waterloo, Waterloo, ON Canada; 4 Program in Medical and Population Genetics, Broad Institute of MIT and Harvard, Cambridge, MA USA

**Keywords:** Gene regulation, Gene expression, Disease genetics

## Abstract

Untranslated regions (UTRs) flank the protein-coding sequence of a gene. 5′UTR and 3′ UTR sequences mediate post-transcriptional regulation via linear and structural elements, controlling RNA stability, cellular localisation and the rate of protein translation. Variants within both 5′ and 3′ UTRs have been shown to cause disease through a variety of diverse mechanisms. However, for these variants to be routinely annotated and interpreted in clinical genetic testing, we need a better understanding of these regions and the spectrum of disease-causing variants within them. In this review, we systematically assess previously identified Mendelian disease-causing variants within UTRs and catalogue their underlying mechanisms. With genome sequencing becoming readily available and increasingly incorporated in diagnostic settings, this review will provide a valuable resource for the consideration and interpretation of UTR variants.

## Introduction

Ascertaining the genetic basis of disease is important in the clinical management of patients, potentially provides therapeutic targets, and informs patients and their families about reproductive decisions. To date, clinical genetics has mostly focused on variants in the regions of the genome that directly encode protein, with associated genetic diagnostic rates for severe rare diseases of only ~30–50% [1]. Although it is known that variants in non-coding regions can also cause disease, this is a comparatively understudied area. Indeed, only recently have recommendations been published to support routine clinical classification of variants in non-coding regions [2].

This review focuses on variants within untranslated regions (UTRs) and their role in Mendelian disease. While UTRs have long been named ‘untranslated’, we now know that regulatory elements within both 5′ and 3′ UTRs undergo active translation. Consequently, these regions may more appropriately be referred to as ‘leader’ (5′UTR) and ‘trailer’ (3′UTR) sequences. However, we use the more familiar UTR terminology here. UTRs are the non-coding regions directly flanking the protein-coding sequence (CDS) of a gene, which are transcribed into mRNA but not translated into the canonical protein. They play an important role in gene regulation and are highly variable across genes [3]. Understanding functional regulatory elements within UTRs is pertinent in understanding their role in translation and function, and how perturbation can cause disease. By increasing knowledge of these regions, we can begin to incorporate analysis of them in clinical genetics and improve diagnostic rates [4, 5]. Hence, here we systematically review regulatory features in both 5′ and 3′ UTRs that, when disrupted, have been shown or hypothesised to cause Mendelian disease. We exclude more common, complex disorders that have been reviewed previously [6, 7].

## UTRs are important regulatory elements

Protein production is a fine-tuned process in cells; too much or too little can disrupt cellular processes and lead to disease. 5′ and 3′ UTRs together play a vital role in the regulation of protein production by controlling transcript stability and the rate and location of protein synthesis.

The primary function of 5′UTRs is in translational regulation. Translation of mRNAs into protein typically begins with ribosome recruitment at the 5′cap of the mRNA, ribosome scanning along the mRNA in a 5′ to 3′ direction (i.e. through the transcript leader), and initiation of translation at a start codon, which is usually an AUG [8]. This process is regulated through key sequence elements such as upstream open reading frames (uORFs) and structural features which influence the amount and speed of protein production, usually by interacting with, or pausing the scanning ribosome [9]. The length of the 5′UTR, as well as the amount and type of various translational control elements within it, varies widely between genes [3], providing each with the correct combination of required elements for normal protein production. Although 5′UTRs are on average very short (mean ~200 bps), in some genes they are longer than the CDS. Typically, genes where careful control of dosage is important have longer and more complex 5′UTRs [3]. Alternative splicing and different transcription start site (TSS) usage can lead to diverse 5′UTR isoforms containing different combinations of translational control elements, enabling different levels of protein translation across tissues and developmental stages [10].

The primary roles of 3′UTRs are in regulating the stability of the mRNA molecule, the rate at which it is degraded, and where it is located within the cell, although, similar to 5′UTRs, they also play a role in the regulation of translation (reviewed by Mayr [11]). Much of this regulation by 3′UTRs is mediated through interactions with additional factors, namely microRNAs (miRNAs) and RNA-binding proteins (RBPs) that bind to motifs and structural elements within 3′UTRs to mediate their effect. While miRNAs are generally repressive, RBPs have a range of diverse regulatory roles (reviewed by Hentze et al. [12]). As with 5′UTRs, the precise combinations of regulatory elements vary widely between genes and between different transcript isoforms of the same gene. Alternative polyadenylation creates distinct transcript isoforms with different length 3′UTRs containing different numbers of binding sites for regulatory miRNAs and RBPs.

**Fig. 1:**
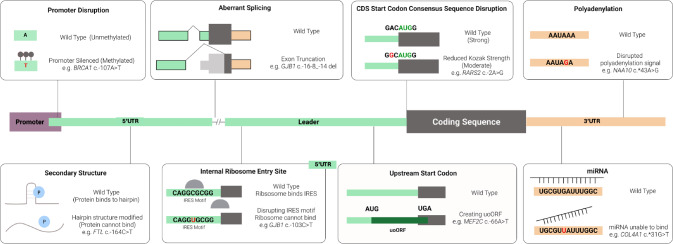
Illustration of UTR regulatory elements and examples of disruptions that lead to disease. Gene and protein expression is a tightly controlled process. Elements on the DNA level (e.g. promoter) and mRNA level (e.g. uAUG codon) influence this process. When these elements are disrupted, they can lead to disease.

Given their crucial roles in gene regulation, perturbing one or more of the regulatory elements within UTRs through genetic variation can have a dramatic impact on protein production and lead to severe disease (Fig. 1). The various underlying mechanisms that have been uncovered to date are reviewed in the following sections. All the variants that are used as examples are listed in Table 1.

**Table 1:** Examples of UTR variants in Mendelian disease.

Region	Mechanism	Example (Variant) GRCh38	Example (Disease)	ClinVar ID	Reference
5′UTR	Promoter disruption through methylation	*BRCA1*:NM_007294.4:c.-107A > T	Breast and ovarian cancer	1685584	[45]
		chr17:43125358-T-A			
5′UTR	Altered Transcription Start Site	*MERTK*:NM_006343.3:c.-125G > A	Inherited retinal disease	*NA*	[5]
		chr2:111898611-G-A			
5′UTR	Aberrant Splicing	*GJB1:*NM_000166.6:c.-16-8_-14del	Charcot-Marie-Tooth Disease	*NA*	[28]
		chrX:71223684_71223694del			
5′UTR	Aberrant Splicing	*PAX6*:NM_001368894.2:c.-128-2del	Aniridia	430969	[27]
		chr11:31806926-CT-C			
5′UTR	Create NTE	*IFITM5*:NM_001025295.3:c.-14C > T	Osteogenesis imperfecta Type V	37143	[22]
		chr11:299504-G-A			
5′UTR	Create NTE	*MEF2C:*NM_002397.5:c.−8C > T	MEF2C haploinsufficiency	1202675	[4]
		chr5:88119613-G-A			
5′UTR	Create uoORF	*MEF2C*:NM_002397.5:c.-66A > T	MEF2C haploinsufficiency	1272072	[4]
		chr5:88823854-T-A			
5′UTR	Create uoORF	*SLC22A*:NM_003060.4:c.-149G > A	Carnitine deficiency	25340	[19]
		chr5:132369824-G-A			
5′UTR	Create uoORF	*NF1:*NM_001042492.3:c.-280C > T	Neurofibromatosis type I	2697306	[25]
		chr17:31095030-C-T			
5′UTR	Create uORF	*TWIST1*:NM_000474.3:c.-263C > A	Saethre-Chotzen syndrome	*NA*	[21]
		chr7:9117584-G-T			
5′UTR	Create uORF	*NIPBL:*NM_133433.3:c.-457_-456delinsAT	Cornelia de Lange syndrome	1300231	[20]
		chr5:36876903-36876904delinsAT			
5′UTR	uoORF removing	*EPHB1*:NM_004441.5:c.-211A > G	Breast and colon cancer	*NA*	[23]
		chr3:134795421-A-G			
5′UTR	uORF removing	*MAP2K6:*NM_002758.4:c.-245T > C	Colon cancer	*NA*	[23]
		chr17:69414740-T-C			
5′UTR	uoORF -> uORF	*THPO:*NM_000460.4:c.-31G > T	Hereditary thrombocythemia	9510	[24]
		chr3:184376290-C-A			
5′UTR	uORF -> uoORF	*NF1*:NM_001042492.3:c.-272G > C	Neurofibromatosis type 1	1379698	[25]
		chr17:31095038-G-C			
5′UTR	uORF -> uoORF	*NF2*:NM_000268.4:c.-66_-65insT	Neurofibromatosis type 2	*NA*	[18]
		chr22:29999922-A-AT			
5′UTR	IRES Disrupting	*GJB1*:NM_000166.6:c.-103C > T	Charcot-Marie-Tooth Disease	217166	[33]
		chrX:71223249-C-T			
5′UTR	IRES Disrupting	*c-MYC:*NM_002467.6:c.577C > T	Multiple myeloma	*NA*	[35]
		chr8:127738794-C-T			
5′UTR	Repeat Expansion	*FMR1*:NM_002024.6:c.-128GGC[200]	Fragile X	183387	[38]
		chrX:147912049-147912050			
5′UTR	Repeat Expansion	*PTCH1:*NM_000264.5:c.-6_-4dup	Basal cell carcinoma	132684	[40]
		chr9:95508364-T-TGCC			
5′UTR	Secondary Structure Altered	*FTL*:NM_000146.4:c.-164C > T	Hyperferritinemia/cataract syndrome	96691	[49]
		chr19:48965344-C-T			
5′UTR	Secondary Structure Altered	*ADAR1*:NM_001111.5:c.-60A > G	Dyschromatosis symmetrica hereditaria	*NA*	[50]
		chr1:154608066-T-C			
5′UTR	CDS start codon consensus sequence altered	*RARS2*:NM_020320.5:c.2T > C	Pontocerebellar hypoplasia	1452675	[52]
		chr6:87589956-A-G			
3′UTR	Disrupted Polyadenylation and cleavage	*NAA10*:NM_003491.4:c.*43A > G	Syndromic microphthalmia	617462	[54]
		chrX:153929948-T-C			
3′UTR	Disrupted Polyadenylation cleavage signal	*F2:*NM_000506.5:c.*97G > A	Thrombophilia	13310	[55]
		chr11:46739505-G-A			
3′UTR	CDS stop codon readthrough affecting poly(A) tail	*HBA1*:NM_000558.5:c.427T > C	α-thalassemia	15624	[56]
		chr16:177409-T-C			
3′UTR	miRNA binding site altered	*COL4A1*:NM_001845.6:c.*31G > T	Cerebral small vessel disease	689433	[59]
		chr13:110150332-C-A			
3′UTR	miRNA binding site altered	*REEP1:*NM_001371279.1:c.808C > T	Hereditary spastic paraplegia type 31	1166866	[60]
		chr2:86217086-G-A			
3′UTR	Repeat Expansion	*DMPK*:NM_004409.5:c.*224CTG[330]	Myotonic dystrophy type 1	810828	[42]
		chr19:45770204-45770205[GCA]			
3′UTR	Repeat Expansion	*ATXN8OS*:NR_002717.2:n.1103CTG[107_127]	Spinocerebellar ataxia type 8	562101	[43]
		chr13:70139384-70139386[CTG]			
3′UTR	Secondary Structure Altered	*SEPN1*:NM_020451.3:c.*1107T > C	SEPN1-related myopathy	844320	[51]
		chr1:25816825-T-C			
3′UTR	Aberrant Splicing	*F8*:NM_000132.4:c.*56G > T	Haemophilia A	1163207	[31]
		chrX:154837541-C-A			

Summary of identified UTR variants, their predicted mode of disruption, and corresponding ClinVar accession IDs.

## Variants that create upstream start codons decrease normal CDS translation

**Fig. 2:**
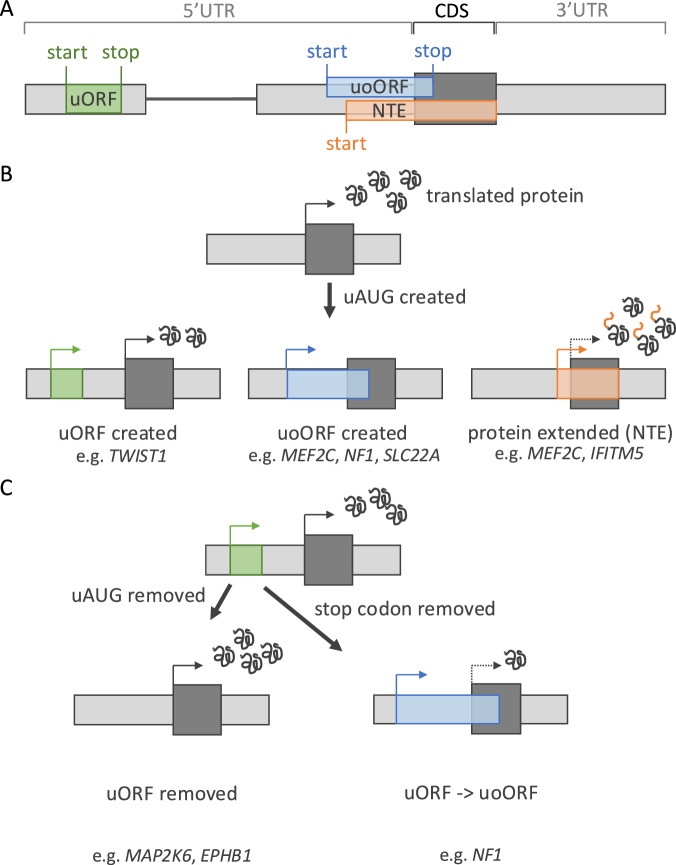
Creation and disruption of upstream open reading frames (uORFs) in disease. **A** Schematic of the different elements encoded by an upstream start codon. uORFs are encoded by an upstream start with an in-frame stop codon also within the 5′UTR. When there is no in-frame stop within the 5′UTR, either an upstream overlapping ORF (uoORF) is formed when the start codon is in a different reading frame to the coding sequence (CDS), or the CDS is extended at the N-terminus (N-terminal extension; NTE). **B** Depiction of the different impacts of variants that create uORFs, uoORFs, or NTEs on CDS translation. **C** Depiction of two types of uORF-perturbing variants on CDS translation. Removing the start codon of a uORF (left) is predicted to increase CDS translation, whereas removing the stop codon of a uORF, resulting in its extension to form a uoORF (i.e. if there is no alternative in-frame stop codon), is predicted to decrease, or abolish, CDS translation.

Upstream AUG (uAUG) triplets are commonly observed within 5′UTRs. uAUGs, or other near-cognate codons (most commonly CUG), may be recognised by the scanning ribosome as start codons which can initiate translation [13]. Translation from an upstream start codon may have one of multiple effects (Fig. 2A). uORFs are encoded when a start codon has an in-frame stop codon before the start of the CDS (i.e. within the 5′UTR). Translation of a uORF can be followed either by ribosome dissociation from the mRNA (therefore decreased CDS translation) or continued scanning before re-initiation of translation at the downstream CDS [14]. If there is no in-frame stop codon, translation from an upstream start would overlap the CDS. If this upstream start is in-frame with the CDS start codon, translation from it will result in an N-terminally elongated protein (N-terminal extension, NTE). Alternatively, if the upstream start is out-of-frame with the CDS, an upstream overlapping ORF (uoORF) will be translated out-of-frame with the CDS, terminating within the CDS or past the CDS stop codon [15, 16]. Translation from upstream start codons is generally assumed to repress CDS translation, as active translation of uORFs has been shown to reduce downstream CDS translation by up to 80% [15]. The prospect of a uAUG initiating translation by influencing the recognition of the start codon by the ribosome is dependent on the local sequence context, known as the Kozak consensus sequence [16]. Around 43% of genes have one or more uAUGs in their 5′UTR [3], and these uAUGs are conserved to a significantly greater degree than any other triplet in 5′UTRs [17]. Creation of new uAUGs has been shown to be under strong negative selection. In particular, variants that create uoORFs or NTEs are, on aggregate, as deleterious as missense variants [18].

Variants that create out-of-frame uoORFs have been found across a range of diseases. This includes the recessive gene *SLC22A*, causing carnitine deficiency (SLC22A:NM_003060.4:c.-149G > A), where multiple patients were compound heterozygous, with one uAUG-creating variant in the 5′UTR and one CDS variant both predicted to reduce protein levels [19]. Other examples include *MEF2C* (NM_002397.5:c.-66A > T) and *NF1* (NM_001042492.3:c.-280C > T), where uoORF-creating variants were found to cause severe developmental disorders [4] and neurofibromatosis type I [18], respectively. In each case, translation of a uoORF leaves the ribosome unavailable to translate the CDS. If the uoORF is translated at high levels due to creation of an upstream start codon into a favourable sequence context, this can cause a complete loss of CDS translation. Conversely, if initiation of translation at the upstream start codon is incomplete, due to a process termed ‘leaky scanning’ [9], the variant will only result in a partial decrease in protein levels. Whether such hypomorphic variants have a large enough effect to cause disease is dependent on the level of dosage sensitivity of each individual gene, complicating variant interpretation.

uORF-creating variants have also been reported to cause disease, again acting to lower CDS translation. Examples include variants in *NIPBL* causing Cornelia de Lange syndrome (NM_133433.3:c.-457_-456delinsAT) [20] and in *TWIST1* (NM_000474.3:c.-263C > A) causing Saethre-Chotzen syndrome [21]. uORF-creating variants are, however, even more difficult to interpret as the effect of introducing a new uORF on CDS translation is hard to predict. Not only does the uAUG context impact the strength of translation of the uORF, but also the length of the uORF created, and the distance from the end of the uORF to the CDS affect the chance of the ribosome re-initiating translation downstream at the CDS start codon. Notably, the interpretation of all uAUG-creating variants is further complicated by the complex makeup of 5′UTRs. For example, if a uAUG is created within an already highly translated uORF, it will likely not be ‘seen’ by scanning ribosomes as a potential start site. Alternatively, creation of a uORF which then prevents translation of an existing uORF with a more repressive effect on translation, could result in up-regulation of CDS translation.

While uORF and uoORF create variants that impact translational regulation, variants that create uAUGs in frame with the CDS that result in NTE may be more likely to disrupt protein function. For example, a variant (NM_001025295.3:c.-14C > T) that extends the N-terminus of *IFITM5* by five amino acids was found recurrently in individuals with osteogenesis imperfecta type V [22]. In this case, the addition of the extra amino acids renders the protein to be non-functional. Similarly, two distinct variants that add three (NM_002397.5:c.−8C > T) and nine (NM_002397.5:c.-26C > T) amino acids, respectively, to the start of the MEF2C protein cause MEF2C haploinsufficiency and severe developmental disorders [4]. *MEF2C* cannot tolerate the addition of amino acids at the N-terminus, as this likely disrupts the binding of this transcription factor to DNA, abolishing its function.

## Variants impact existing uORFs or uoORFs can disrupt translational regulation

The above examples of uAUG-creating variants are all predicted to decrease CDS translation. Conversely, removing an existing upstream start codon is more likely to result in an increase in protein levels and have a gain-of-function effect. Upstream start-codon removing variants in *EPHB1* (breast and colon) (NM_004441.5:c.-211A > G) and *MAP2K6* (colon) (NM_002758.4:c.-245T > C) in cancer samples (removing an uoORF and uORF, respectively) were found to be associated with enhanced translation, suggesting that loss-of-uAUG mediated translational increase of the downstream main protein-coding sequence may contribute to carcinogenesis [23].

Variants may also alter the inhibitory effect of uORFs or uoORFs. For example, a variant in the 5′UTR of *THPO* (NM_000460.4:c.-31G > T) turns a uoORF into a uORF through the creation of a stop codon within the uoORF sequence. The native uoORF is strongly translated, leading to a necessary low production of *THPO. THPO* encodes thrombopoietin, required for normal functioning of the pathway controlling the production of platelets. Turning the uoORF into a uORF (with the possibility of re-initiation) increases protein levels, causing hereditary thrombocythemia [24]. Contrastingly, changing a highly translated uORF into a uoORF can increase its inhibitory effect. This can be achieved through two different mechanisms. An example of the first is seen in *NF1*, where variants (e.g. NM_001042492.3:c.-272G > C) that remove the stop codon of a native uORF, transforming it into an uoORF (as there are no alternative in-frame stop codons before the CDS), have been observed in patients with neurofibromatosis type I [25]. The second mechanism is seen in *NF2*, the 5′UTR of which contains a native uORF with prior evidence of translation and a strong predicted Kozak consensus. A single base insertion (NM_000268.4:c.-66_-65insT) changes the frame of the uORF, causing it to bypass the downstream stop codon and create an out-of-frame uoORF. Translation of this uoORF is predicted to lower translation of *NF2*, consistent with haploinsufficiency causing neurofibromatosis type 2 [18]. Similarly to uAUG-creating variants, interpretation of variants that alter existing uORFs can be complex. The difference in translational repression conveyed by uORFs and uoORFs, and uORFs of different lengths, is difficult to predict.

## Variants can cause disease by altering UTR splicing

**Fig. 3:**
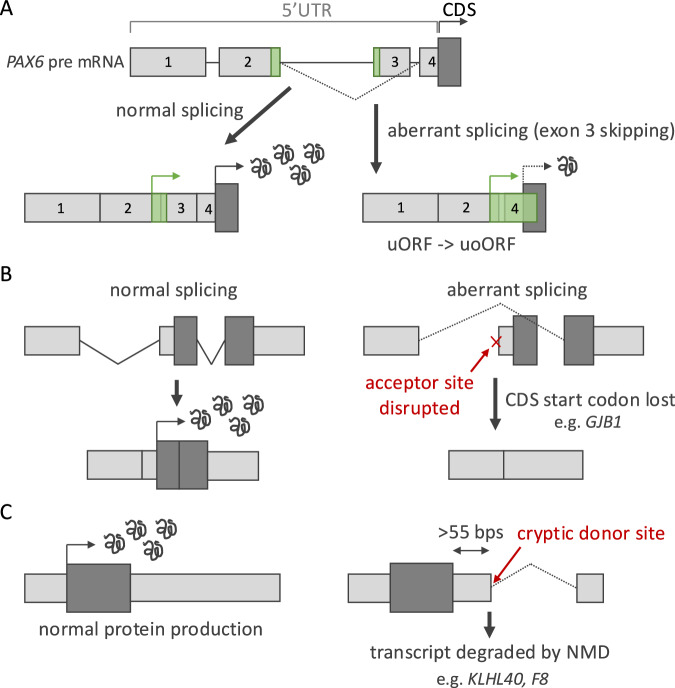
Schematic showing how UTR variants can cause disease through disrupting RNA splicing. **A** 5′UTR variants in *PAX6* are a common cause of aniridia. These variants are thought to cause skipping of exon three, which contains the stop codon of an upstream open reading frame (uORF), converting it into an upstream overlapping ORF (uoORF) and resulting in lower translation of the *PAX6* coding sequence (CDS). **B** Variants that disrupt the acceptor splice site of the final exon in the 5′UTR remove the start codon of the CDS. **C** 3′UTR variants that create cryptic donor splice sites further than 55 bps downstream of the end of the CDS are predicted to lead to transcript degradation through nonsense-mediated decay (NMD).

Variants within UTRs that interfere with splicing can cause disease through a variety of mechanisms (Fig. 3). Approximately 38% of 5′UTRs contain introns, with the number of introns ranging to 11 [3]. Alternative splicing of 5′UTRs is known to impact mRNA stability and translation [26].

Several variants affecting splicing within the 5′UTR of *PAX6* are considered pathogenic for aniridia [27]. These variants (e.g. NM_001368894.2:c.-128-2del) induce exon skipping or splicing errors around exons 2 and 3 of the 5′UTR. The hypothesised mechanism of disease is through uORF dysregulation (Fig. 3A); there is a uORF crossing these exons, and the variants reportedly change the uORF frame, which, similar to the *NF2* example above, turns the uORF into a more inhibitory uoORF, leading to loss-of-function and disease [27].

Altering 5′UTR splicing can also impact the CDS sequence. For example, variants that disrupt the final acceptor site in the 5′UTR can lead to skipping or truncation of the exon containing the canonical CDS start codon. One such example is in *GJB1* (NM_000166.6:c.-16-8_-14del), where this mechanism causes a significant amount (278 bps) of the following exon to be removed, including 262 bps of the CDS (31%; Fig. 3B). This variant is reported to lead to Charcot-Marie-Tooth disease [28].

While splicing is common in 5′UTRs, only around 6% of 3′UTRs contain introns [29]. The vast majority of these introns are very close to the CDS stop codon, as introns >50/55 bps downstream of the CDS would be predicted to cause transcript degradation through the nonsense-mediated decay (NMD) pathway [30]. Variants that create new introns into 3′UTRs can trigger NMD and result in loss of protein expression. An example is a variant in *F8* (NM_000132.4:c.*56G > T) that creates a new donor splice site, resulting in a 159 bp deletion in the 3′UTR, demonstrated to reduce expression and therefore thought to cause mild haemophilia A [31].

## Variants in internal ribosome entry sites (IRES) may impact ribosomal recruitment

A subset of mRNAs can initiate translation in a cap-independent manner via internal ribosome entry sites (IRESs). These are specialised sequences within 5′UTRs that can directly recruit 40s ribosomal subunits to initiate scanning from within the 5′UTR independently of the 5′cap [32]. Notably, IRES motifs differ between genes so are difficult to predict, limiting our ability to annotate and interpret variants in these elements. In addition, while there are documented examples of IRES in many organisms, the evidence supporting their widespread existence in humans is limited. Potentially as a consequence, there are very few mentions of IRES’s role in disease in the published literature. Exceptions include a variant (NM_000166.6:c.-103C > T) in *GJB1* segregating with Charcot-Marie-Tooth disease. The native IRES of *GJB1* is essential for translation of connexin-32 mRNA in nerve cells, and the variant in this case was reported to abolish the IRES’s function, leading to no translation, as was discovered via in-depth in vivo analysis using bicistronic reporters [33]. However, the existence of this IRES was thrown into doubt by more recent experimental work [34]. A further example is in the proto-oncogene c-myc, where in patients with multiple myeloma, a C > T substitution (NM_002467.6:c.577 C > T) within a reported IRES causes higher IRES activity and increased production of c-myc protein. The underlying mechanism is not fully understood, but experimental data suggested that c-myc IRES trans-acting factors (Y-box binding protein 1 (YB-1) and polypyrimidine tract-binding protein 1 (PTB-1)) bind more strongly to the mutated version of c-myc IRES [35]. Of note, newer transcript annotations categorise this variant as a missense variant within the CDS.

## Repeat expansions in UTRs can disrupt regulation and cause toxic peptide production

Repeat expansions (also known as microsatellites or simple sequence repeats) are a unique class of variation. The nucleotide sequence, location within the gene, range of repeat length and clinical outcomes vary between repeats. Repeat expansions can be pathogenic when located in non-coding regions, causing so-called non-coding repeat expansion disorders [36], with many examples within UTRs. The pathogenic mechanisms vary, but the main possible mechanisms are as follows (reviewed in [37]): repeat sequences can form intramolecular structures that can influence transcription, translation and binding to various RBPs; GC-rich repeat sequences are also prone to hypermethylation that can cause gene silencing; more rarely, through repeat associated non-ATG (RAN) translation, the repeat RNA itself might be unconventionally translated into toxic peptides.

CGG repeat expansions in the 5′UTR of *FMR1* (NM_002024.6:c.-128GGC[200]) lead to hypermethylation and silencing of the gene. This results in insufficient amounts of FMR1 protein that is required for neuronal development and causes Fragile X syndrome [38]. A CGG repeat expansion in the *GIPC1* 5′UTR is associated with oculopharyngodistal myopathy. The number of CGG repeats is <30 in controls but >60 in affected individuals [39]. However, genes can be even more sensitive to the number of repeats; insertion of a surplus eighth CGG triplet in the *PTCH1* 5′UTR (NM_000264.5:c.-6_-4dup) represses protein translation drastically compared to the wild-type seven-time repeat sequence. This non-coding variant is reported to predispose to basal cell carcinoma [40].

There are also multiple instances of pathogenic repeat expansions within 3′UTRs. For example, a large CTG repeat (NM_004409.5:c.*224CTG[330]) in the 3′UTR of *DMPK* causes myotonic dystrophy type 1 (DM1) via a toxic gain-of-function mechanism [41]. It is hypothesised that the mutant *DMPK* transcripts form aberrant structures and anomalously associate with RBPs [42]. Similarly, spinocerebellar ataxia type 8 (SCA8) can be caused by a CTG expansion (NR_002717.2:n.1103CTG[107_127]) in the 3′UTR of *ATXN8OS* [43]. Here, there is evidence for both toxic RNA and toxic protein effects, as RAN translation can generate a polyglutamate protein from the antisense strand [37].

## 5′UTR variants that overlap the promoter can disrupt transcription initiation

Promoters are sequences of DNA that initiate transcription of a gene. Relevant proteins, termed transcription factors, bind to promoter motifs to initiate transcription [44]. Promoters typically span the TSS of a gene, which also marks the beginning of the 5′UTR. Hence, the portion of the promoter that is downstream of the TSS may also overlap the 5′UTR. A variant (NM_007294.4:c.-107A > T) in the 5′UTR-overlapping promoter region of *BRCA1* observed in two unrelated families is associated with epigenetic silencing through allele-specific promoter methylation and is thought to lead to grade 3 breast cancer or high-grade serous ovarian cancer [45]. However, another study questions this: the authors looked for this variant in a larger cohort of patients with *BRCA1* allele-specific promoter methylation and did not find this variant [46]. Another example is a variant (NM_006343.3:c.-125G > A) reported to overlap the TSS of *MERTK* was hypothesised to disrupt transcription, alter secondary structure and cause inherited retinal disease [5]. This was supported by a reduction in mRNA levels in a luciferase reporter assay.

## Variants can cause disease by disrupting mRNA secondary structure

mRNA is a single-stranded RNA sequence capable of folding and forming secondary structures through complementary base-pairing with itself. These structures can impact translation by causing inefficient ribosomal scanning and affecting mRNA stability [47]. There are several types of secondary structures, including pseudoknots, G-quadruplexes and hairpin structures (also referred to as stem-loops), which occur when the mRNA strand folds and base pairs with an adjacent section. It is difficult to predict the exact conformation and structure of mRNA based on sequence alone and secondary structures are often dynamic.

One of the best-studied examples of an important stem-loop is the iron-responsive element (IRE), which affects translation of mRNAs important for iron homeostasis. Cellular iron uptake must be tightly regulated, as insufficient or excess levels of iron can be damaging. A single conserved IRE stem-loop close to the 5′cap of mRNA is bound by iron-regulatory protein 1 or 2 (IRP1/IRP2). IRP binding represses translation initiation by blocking ribosome access to the 5′UTR. The IRPs register iron availability through direct interactions with iron in the cytosol and alter their binding and hence translational inhibition in response [48]. L-ferritin binds and stores iron in the cell. Hyperferritinemia/cataract syndrome (HHCS) is caused by mutations (e.g. NM_000146.4:c.-164C  >  T) in the IRE in the 5′UTR of the L-ferritin (*FTL*) gene, preventing interaction with the IRPs and leading to dysregulated high levels of L-ferritin production [49].

Another example of altering mRNA secondary structure involves a heterozygous variant (NM_001111.5:c.-60A > G) in the 5′UTR of *ADAR1* that reduces gene expression and is reported to cause dyschromatosis symmetrica hereditaria. The variant does not appear to disrupt any known regulatory features in that region; however, this single-nucleotide change is thought to be sufficient to alter the structure of the mRNA [50]. Exactly how this structural change results in reduced gene expression is not currently known.

SEPN1-related myopathy consists of four autosomal recessive disorders. *SEPN1* produces selenoprotein, which is required for normal muscle development. A unique feature of all selenoproteins is the presence of the amino acid selenocysteine, unusually encoded by the CDS stop codon, UGA. This re-coding of UGA is made possible by a highly-conserved stem-loop structure that starts 6 bp downstream of the CDS UGA codon, in the 3′UTR. This region is known as the selenocysteine insertion sequence (SECIS). A SECIS RBP (SBP2), which binds SECIS, is vital in the redefinition of the UGA to a selenocysteine codon, preventing termination at the UGA. Termination at the UGA triggers NMD and leads to insufficient protein. Three variants (e.g. NM_020451.3:c.*1107T > C) specifically within the stem-loop structure of the SECIS have been linked to SEPN1-related myopathy by interfering with SBP2 binding, significantly reducing both mRNA and protein levels [51].

## 5′UTR variants can alter the efficiency of translation initiation at the CDS start codon

The exact context surrounding the CDS start codon dictates the strength of translation from that AUG and hence the amount of protein produced. Variants within the 5′UTR that disrupt this Kozak consensus sequence can influence disease risk. A recent paper by Nicolle et al. describes a variant in the 5′UTR of *RARS2* (NM_020320.5:c.2T > C) which alters the Kozak sequence of the CDS start codon, reducing protein production and causing pontocerebellar hypoplasia (PCH) [52]. The authors also assessed ClinVar for other variants that are predicted to alter the Kozak sequence of the CDS and found 20, most of which are denoted as variants of unknown significance. They conclude that variants of this class are likely an underappreciated mechanism of disease.

## Variants in 3′UTRs can affect polyadenylation

For most protein-coding genes, the 3′ end of the pre-mRNA is formed by consecutive cleavage and polyadenylation that occurs co-transcriptionally. This is a widely studied phenomenon and is reviewed by Curinha et al. [53] and summarised here. 3′UTRs contain a polyadenylation site (polyA site (PAS)) that directs the addition of several hundred adenine residues to create the polyA tail at the end stage of transcription. The polyA tail has an important role in nuclear export, translation and the stability of the mRNA. Usage of a specific PAS in the pre-mRNA is directed by RNA cis-elements and several trans-acting factors. The most important cis-element is the polyA signal, a hexamer (usually AAUAAA or a close variant such as UAUAAA) located ~10–35 bp upstream of the PAS.

Multiple variants affecting polyadenylation are associated with disease [53]. For example, several variants within the PAS of *NAA10* (e.g. NM_003491.4:c.*43A > G) have been linked to syndromic X-linked microphthalmia. In vitro studies demonstrated that these variants disrupt cleavage and polyadenylation and lead to reduced mRNA levels [54]. Conversely, a gain-of-function single-nucleotide variant at the terminal end of the *F2* 3′UTR (NM_000506.5:c.*97G > A) causes elevated prothrombin plasma levels. The wild-type polyadenylation cleavage signal is inefficient, but the variant increases cleavage site recognition, increases 3′ end processing, mRNA accumulation and protein synthesis, leading to thrombophilia [55].

While not strictly a 3′UTR variant, a single-nucleotide variant that changes the α-globin (*HBA1*) CDS stop codon from a UAA to CAA (NM_000558.5:c.427T > C) allows the translating ribosomes to proceed into the 3′UTR. This is associated with a substantial decrease in α-globin mRNA half-life. Further experiments elucidated that there are C-rich regions that interact with a ribonucleoprotein complex, the α-complex, containing proteins that prevent degradation of the mRNA. The α-complex is thought to protect the poly(A) tail and stabilise the mRNA. If this interaction is prevented, such as in this case, the poly(A) tail undergoes accelerated shortening; the mRNA is prematurely degraded and α-thalassemia ensues [56].

## Variants disrupting microRNAs and their binding sites alter RNA silencing

miRNAs are non-coding RNAs ~22 bp long which bear one or more hairpin loops. They are involved in RNA silencing and post-transcriptional regulation of gene expression. miRNAs base pair to complementary sequences on the mRNA, usually within 3′UTRs, and can silence and inhibit protein production, for example, through mRNA deadenylation and decapping [57].

There are multiple examples of pathogenic variants within miRNAs themselves, specifically within the ‘seed region’ that is responsible for mediating mRNA binding [58]. In addition, variants in miRNA-binding sites within 3′UTRs can disrupt miRNA-mediated regulation. For example, variants within a 7 bp region of the *COL4A1* 3′UTR (e.g. NM_001845.6:c.*31G > T) abolish a binding site for the miR-29 miRNA, leading to increased levels of *COL4A1* mRNA and leading to cerebral small vessel disease [59]. Conversely, two variants in the miR-140 binding site in the 3′UTR of *REEP1* (e.g. NM_001371279.1:c.808C > T) are predicted to suppress miRNA-mediated effects on translation, leading to a decrease in REEP1 protein. This causes hereditary spastic paraplegia type 31 [60].

## Discussion

Here, we systematically reviewed important regulatory elements in 5′ and 3′ UTRs that, when perturbed, can cause Mendelian disease. These regions have historically been overlooked, but with the advancement and inclusion of whole-genome sequencing in clinical diagnostic sequencing, these regions are becoming more accessible. Recent recommendations for clinical interpretation of non-coding variants [2] aid in classifying UTR variants in a clinical setting; however, annotating and interpreting these variants remains a considerable challenge.

Annotating variants within UTRs currently requires using a range of bioinformatics tools and datasets. Each tool can annotate variants according to a particular hypothesised effect, for example, UTRannotator [61], but there is no single solution that combines all known regulatory elements and variant mechanisms. Each variant may also have multiple predicted effects, for example, creating a uAUG and altering a transcription regulatory factor binding site [62]. Determining exactly how a variant is mediating an effect on protein expression can be difficult without extensive functional characterisation.

Currently, UTR variants are most often annotated with respect to known regulatory elements; however, these elements may be incompletely annotated, especially if they are tissue or temporally specific. In addition, there may be further, as yet unknown categories of regulatory elements through which variants may mediate their effect. This is highlighted by reportedly disease-causing UTR variants that act via unknown mechanisms. An example is a 96 bp deletion in the 3′UTR of *VMA21* that segregates with X-linked myopathy with excessive autophagy (XMEA) and was demonstrated to reduce mRNA quantity. The underlying mechanism is unknown but may involve destabilisation of the mRNA [63].

A key challenge in interpreting UTR variants, and non-coding variants more broadly, is that they often have incomplete, or hypomorphic, effects. In addition, these effects can be in either direction, resulting in either an increase or a decrease in protein levels, as demonstrated by many of the variants detailed above. For example, uoORF-creating variants in *MEF2C* decrease protein expression [4] and variants in the *THPO* 5′UTR increase protein expression [24], both leading to respective diseases. The threshold at which increased or decreased protein expression leads to disease is highly gene-specific, but these thresholds are not known for the vast majority of genes. For partial effect non-coding variants that do cause disease, they may result in milder phenotypes, for example, variants in the CDS of *KLHL40* are linked to severe forms of nemaline myopathy [64], whereas a variant in the 5′UTR of *KLHL40* is linked to a milder form of disease [65]. Additionally, partial effect non-coding variants that do cause disease may also result in later disease onset and/or have reduced penetrance [66].

Interpretation of UTR variants is also complicated by the need to consider wider sequence context. UTRs are complex regulatory elements with precise combinations of regulatory elements. Similarly to the redundancy observed in enhancers [67], other regulatory elements may be able to compensate if one element is disrupted by a genetic variant. This may be the case for miRNA-binding sites, for example, where multiple binding sites for the same miRNA can be found within the same 3′UTR [68]. Consideration of wider sequence context is also critical in deciphering the impact of 5′UTR variants that disrupt translational regulation. For example, not all upstream start codon creating variants are created equal: the chance of translation from an upstream start is dependent not only on the surrounding sequence match to the Kozak consensus, but also on the position of other translated uORFs [69]. For example, if the start is created within a ubiquitously translated uORF it will not be ‘seen’ by a scanning ribosome and hence will not have any impact on downstream CDS translation.

Given the challenges in the interpretation of UTR variants, functional characterisation is important. In addition to experiments to decipher the effect of individual variants observed in patients, large-scale multiplexed assays of variant effect hold great promise not only for variant interpretation but also for us to learn more about gene regulation [70]. Importantly, however, variants in UTRs can act through multiple different mechanisms: impacting transcript, RNA-processing, RNA stability and translation. A variant impact cannot be ruled out unless all these mechanisms have been captured in the experiments performed [2]. Alternatively, if an assay focuses on a downstream impact such as cell viability, the exact mechanism of effect may remain unclear. Further, as discussed above, UTRs act as entire functional elements. Including the entire sequence in its native context is likely important for accurate functional characterisation.

Variants in 5′ and 3′ UTRs are a rare but underappreciated cause of Mendelian disease. Annotating and interpreting these variants in clinical settings is challenging but can result in a diagnosis for patients and, in turn, increase our knowledge of UTR-mediated gene regulation.
